# Intracranial peak pressure as a predictor for perioperative mortality after spontaneous intracerebral hemorrhage evacuation and decompressive craniectomy

**DOI:** 10.1186/s41016-023-00316-5

**Published:** 2023-01-18

**Authors:** Zhong Wang, Ruijian Zhang, Zhitong Han, Yisong Zhang, Junqing Wang, Bo Wang, Baiyu Liu, Weiran Yang

**Affiliations:** Department of Neurosurgery, Inner Mongolia People’s Hospital, 20 ZhaoWooda Road, Hohhot, Inner Mongolia People’s Republic of China

**Keywords:** Intracranial pressure, Intracerebral hemorrhage, Decompressive craniectomy, Outcomes, Perioperative mortality

## Abstract

**Background:**

An optimal intracranial pressure (ICP) management target is not well defined in patients with spontaneous intracerebral hemorrhage. The aim of this study was to explore the association between perioperative ICP monitoring parameters and mortality of patients with spontaneous intracerebral hematoma undergoing emergency hematoma removal and decompressive craniectomy (DC), to provide evidence for a target-oriented ICP management.

**Methods:**

The clinical and radiological features of 176 consecutive patients with spontaneous intracerebral hemorrhage that underwent emergent hematoma evacuation and DC were reviewed. The Glasgow Coma Scale (GCS) and Glasgow Outcome Scale (GOS) scores were assessed 2 weeks after surgery. Multivariate logistic regression analysis was performed to identify predictors for perioperative death.

**Results:**

Forty-four cases (25.0%) were assigned to the ICP group. In patients with an ICP monitor, the median peak ICP value was 25.5 mmHg; 50% of them had a peak ICP value of more than 25 mmHg. The median duration of ICP > 25 mmHg was 2 days. Without a target-specific ICP management, the mortality at 2 weeks after surgery was similar between patients with or without an ICP monitor (27.3% versus 18.2%, *p* = 0.20). In multivariable analysis, the peak ICP value (OR 1.11, 95% CI 1.004–1.234, *p* = 0.04) was significantly associated with perioperative death in the ICP group. The area under ROC curve of peak ICP value was 0.78 (95%CI 0.62–0.94) for predicting mortality, with a cut-off value of 31 mmHg.

**Conclusion:**

Compared with a persistent hyperintracranial pressure, a high ICP peak value might provide a better prediction for the mortality of patients with spontaneous intracerebral hemorrhage evacuation and DC, suggesting a tailored ICP management protocol to decrease ICP peak value.

## Background

Spontaneous intracerebral hemorrhage is a major cause of death and disability all over the world [1]. Emergency hematoma removal with decompressive craniectomy (DC) surgery has been widely used in these patients as a primary intervention, or as an auxiliary intervention when first-line treatment fails [1–3]. An optimal intracranial pressure (ICP) management is critical in perioperative care. Several studies have indicated that DC can relieve hyperintracranial pressure and enhance cerebral perfusion, which can improve long-term neurofunctional outcomes [4–6]. However, two previous randomized clinical trials have failed to demonstrate that DC can improve the prognosis at 6 months after traumatic brain injury [7–9]. In addition, several studies have put forward opposite conclusions on the effectiveness of ICP monitoring [10–12]. Therefore, whether ICP real-time monitoring can improve the prognosis of patients undergoing DC is still controversial, and importantly, it might be the target-specific ICP management that makes the difference beyond ICP monitoring. In this study, we mainly focused on the value of different parameters in perioperative ICP monitoring for predicting mortality of patients with spontaneous intracerebral hematoma undergoing emergent hematoma removal and DC, to identify potential ICP target for improving prognosis.

## Methods

### Study design and participants

The patients included in this study were from a single-center cohort of patients with spontaneous intracerebral hemorrhage between 2017 and 2021. Consecutive cases involving patients with spontaneous intracerebral hemorrhage underwent emergent hematoma evacuation surgery and DC at our institute were reviewed to find those with intraoperative ICP monitoring fiberoptic catheter implantation. Written informed consent for collecting clinical information and radiological data was obtained from each patient at admission. The study was performed according to the guidelines of the Declaration of Helsinki and was approved by the ethics committee of our institute. The inclusion criteria were as follows: (1) a diagnosis of spontaneous intracerebral hematoma confirmed with emergency head computed tomography (CT), and (2) patients who underwent emergency hematoma removal and DC. The exclusion criteria were as follows: (1) patients with clotting dysfunction, (2) patients with severe underlying diseases, and (3) patients who discontinued or abandoned treatment for various reasons after surgery.

Finally, a total of 176 patients were enrolled in this study. According to whether the ICP monitoring fiberoptic catheter was inserted, it was divided into two subgroups: ICP group and non-ICP group.

### Surgical procedure and postoperative management

For the ICP group, all patients underwent standardized emergency DC. Surgical procedure: Making a large, unilateral, curvilinear incision in the frontotemporoparietal region. Then prepare a myocutaneous flap and a free frontotemporoparietal bone flap (12 × 15 cm) for craniectomy. And then, the dura was cut radially, the hematoma was gently removed, and necrotic, contused brain tissue was gently suctioned out. After rigorous hemostasis, the ICP monitoring fiberoptic catheter (Codman, USA) was inserted in the cortex to permit continuous measurement of ICP and the dura mater was sutured by reducing tension. Postoperative dehydration strategy: The upper limit of ICP warning value was set to ICP value ≥ 25 mmHg, and the duration was more than 1 h. Once the warning value was reached, drug treatment (mannitol or hypertonic brine) was given to reduce ICP in time.

For the non-ICP group, except for the implantation of the ICP monitoring fiberoptic catheter, the subsequent surgical procedures were exactly the same as in the ICP group. Postoperative dehydration strategy: Intravenous infusion of 20% mannitol (250 ml/8 h). Routine monitoring of blood pressure, if systolic blood pressure > 220 mmHg or diastolic blood pressure > 120 mmHg, adjust the mannitol dose based on clinical experience, postoperative clinical manifestations, and review of CT images.

Other pre- and postoperative management were identical in both groups and were in accordance with the principles described in the AHA/ASA guidelines for the management of spontaneous intracerebral hemorrhage (2015 Edition) [13].

### Data collection and definition

Patient baseline demographic, clinical features and imaging data were collected. According to the location of the hematoma, the hemorrhage was divided into basal ganglia and/or thalamus hematoma with or without intraventricular hematoma. The preoperative state of consciousness was evaluated using the Glasgow Coma Scale (GCS) system. The threshold for subgroup grouping in ICP group is 25 mmHg.

Two weeks after surgery was set as the time point for perioperative evaluation. The GCS score, Glasgow Outcome Scale (GOS) score, and mortality were compared between these two subgroups [14, 15]. The evaluation of GCS and GOS scores was conducted by neurosurgeons who have at least 5 years’ experience of clinical practice and all the images were interpreted by at least 2 radiologists independently who are with at least 5 years of clinical experience in radiology center of our institute.

### Statistical analysis

The categorical variables are presented as counts (with percentages); the continuous variables are presented as the means ± standard deviations (SD). The Pearson chi-square test or Fisher exact test was used to compare categorical variables as appropriate. Two-tailed *t*-tests were employed to compare continuous variables (normal distribution variable). Wilcoxon rank sum test was applied to compare non-normal distribution continuous variables. Odds ratios (ORs) and 95% confidence intervals (CIs) for predictors of perioperative death were calculated by univariate and multivariate logistic regression analyses. The sensitivity and accuracy of the prediction model to predict prognosis were calculated using the receiver operating characteristics (ROC) curve and the cut-off values of the continuous variables were calculated. *P* value < 0.05 was considered to be statistically significant. Statistical analysis was performed using SPSS (version 25.0, IBM).

## Results

### Baseline characteristics

A total of 176 patients (44 with ICP and 132 without ICP) were included in this study (Table 1). The mean age was similar between the ICP group and non-ICP group (50.5 ± 13.6 years vs 54.6 ± 12.2 years, *p* = 0.060). The preoperative GCS score was 6.2 ± 3.1 (5.9 ± 3.3 vs 6.3 ± 3.1, *p* = 0.462). Most of them (79.5%) were evaluated as GCS $\le$ 6 (81.8% vs 78.8%, *p* = 0.666).

**Table 1:** Baseline characteristics of the study population

Characteristics	ICP group	Non-ICP group	*P* value
No. of patients	44	132	
Age, year			
Mean ± SD	50.5 ± 13.6	54.6 ± 12.2	0.060
Sex			0.025^*^
Male	40 (90.9)	99 (75.0)	
Female	4 (9.1)	33 (25.0)	
Hypertension history			0.333
Yes	28 (63.6)	73 (55.3)	
No	16 (36.4)	59 (44.7)	
Location of the hematoma			
Basal ganglia and/or thalamus hematoma	28 (63.6)	84 (63.6)	1.000
Intraventricular hematoma	12 (27.3)	23 (17.4)	0.156
Preoperative GCS score			
Mean ± SD	5.9 ± 3.3	6.3 ± 3.1	0.462
Mild (GCS 9–10)	4 (9.1)	10 (7.6)	1.000
Moderate (GCS 7–8)	4 (9.1)	18 (13.6)	0.430
Severe (GCS $\le$ 6)	36 (81.8)	104 (78.8)	0.666
ICP characteristics			
Peak ICP value (median, quartile)	25.5 (20, 36)	-	
Peak ICP > 25 mmHg	22 (50.0)	-	
Duration of ICP > 25 mmHg, days (median, quartile)	2 (2,3)	-	

Values are expressed as number of cases (%) or the mean ± standard deviation, unless otherwise indicated

*GCS* Glasgow Coma Scale, *ICP* Intracranial pressure, *SD* Standard deviation

^*^Statistical significance (*p* < 0.05)

In the ICP group, the median peak ICP value was 25.5 (IQR 20, 36) mmHg. Fifty percent have a peak ICP value greater than 25 mmHg. The median duration of ICP > 25 mmHg was 2 (IQR 2, 3) days. The mean length of monitoring was 5 days (range 4–7 days).

### Short-term outcomes

At 2 weeks after the operation, there was no statistically significant difference in mortality between the ICP group and non-ICP group (27.3% vs 18.2%, *p* = 0.195) (Table 2). The mean GCS score was similar (9.6 ± 5.0 vs 9.8 ± 4.2, *p* = 0.749). The prognosis of the ICP group was polarized, most of them were mild coma (31.8%) or severe coma (31.8%). However, the distribution of the short-term prognosis of the non-ICP group was more even. 29.0% of them have deteriorated GCS scores at 2 weeks after the operation compared with admission. The GOS score of the ICP group was 2.6 ± 1.6, which means that most of the patients have severe deficits. The disability rate in the ICP group was 77.3%.

**Table 2:** Postoperative outcomes between patients with or without ICP monitor

Characteristics	ICP group (*n* = 44)	Non-ICP group (*n* = 132)	*P* value
Postoperative outcomes			
GCS score			
Mean ± SD	9.6 ± 5.0	9.8 ± 4.2	0.75
Mild (GCS 9–10)	14 (31.8)	36 (27.3)	0.56
Moderate (GCS 7–8)	4 (9.1)	33 (25.0)	0.03^*^
Severe (GCS $\le$ 6)	14 (31.8)	39 (29.5)	0.78
Death	12 (27.3)	24 (18.2)	0.20
GCS score deteriorated	16 (36.4)	35 (26.5)	0.21
GOS score			
Mean ± SD	2.6 ± 1.6	2.8 ± 1.3	0.49

Values are expressed as number of cases (%) or the mean ± standard deviation, unless otherwise indicated

*GCS* Glasgow Coma Scale, *GOS* Glasgow Outcome Scale, *ICP* Intracranial pressure, *SD* Standard deviation

^*^Statistical significance (*p* < 0.05)

### Predictors for perioperative death in the ICP group

**Fig. 1:**
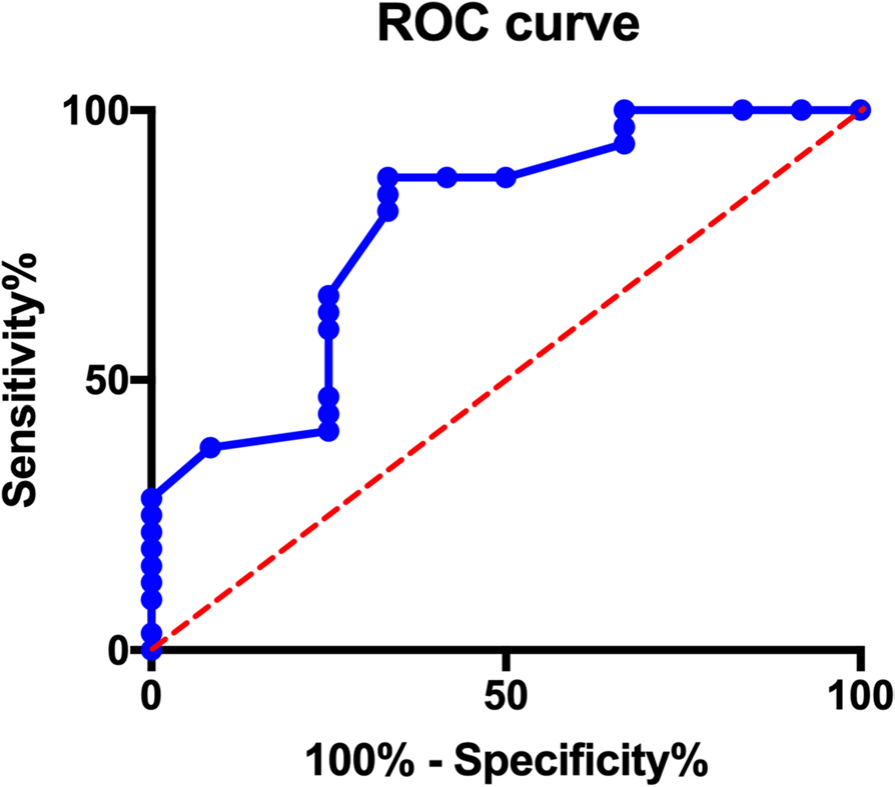
Receiver operating characteristic (ROC) analyses for predicting perioperative death using peak ICP value. ROC curve of peak ICP value for predicting perioperative death (blue curve) (reference line shown in red dashed line)

During 2 weeks after the operation in the ICP group, 12 patients (27.3%) died. In the univariate analysis, peak ICP value (*p* = 0.001) and peak ICP > 25 mmHg (*p* = 0.042) showed a significant association with perioperative death. The duration of ICP > 25 mmHg has a marginal effect (*p* = 0.056). The multivariate logistic regression analysis demonstrated that peak ICP value (OR 1.113, 95% CI 1.004–1.234, *p* = 0.042) was significantly associated with perioperative death in the ICP group (Table 3). ROC curve showed that the AUC of the peak ICP value was 0.784 (95%CI 0.624–0.944). The cut-off value of the peak ICP value was 31 mmHg (Fig. 1).

**Table 3:** Risk factor for perioperative death in the ICP group

Characteristics	Univariate		*P* value	Multivariate		*P* value
	Dead	Survival		OR	95% CI	
No. of patients	12	32				
Age	55.2 ± 8.8	48.8 ± 14.8	0.16			
Sex, male	11 (91.7)	29 (90.6)	0.92			
Peak ICP value	46.2 ± 26.3	25.5 ± 9.9	0.001^*^	1.11	1.004–1.23	0.04^*^
Peak ICP > 25 mmHg	9 (75.0)	13 (40.6)	0.04^*^	0.73	0.034–15.51	0.84
Duration of ICP > 25 mmHg	1.8 ± 1.5	0.9 ± 1.3	0.06	0.85	0.32–2.30	0.75
Hypertension history			0.65			
No	5 (41.7)	11 (34.4)				
Yes	7 (58.3)	21 (65.6)				
Location of the hematoma						
Basal ganglia and/or thalamus hematoma	5 (41.7)	23 (71.9)	0.06			
Intraventricular hematoma	3 (25.0)	9 (28.1)	0.84			
Preoperative GCS score						
Mean ± SD	5.2 ± 3.5	6.2 ± 3.3	0.38			
Mild coma	10 (83.3)	26 (81.3)	Ref			
Moderate coma	1 (8.3)	3 (9.4)	0.92			
Severe coma	1 (8.3)	3 (9.4)	0.92			

Values are expressed as number of cases (%) or the mean ± standard deviation, unless otherwise indicated

*GCS* Glasgow Coma Scale, *ICP* Intracranial pressure, *SD* Standard deviation

^*^Statistical significance (*p* < 0.05)

## Discussion

The primary purpose of cerebral hemorrhage surgery is craniotomy decompression, followed by the removal of hematoma to relieve local mass effect [16]. However, the effect of DC on the prognosis of severe cerebral hemorrhage remains controversial [4–9]. It should be noted that ICP monitoring is one of the core indicators of perioperative treatment of patients with cerebral hemorrhage. In this study, we summarized 44 patients with ICP monitoring and 132 patients without ICP monitoring in our institution. Without an identified ICP management target, we found no statistically significant differences in perioperative mortality between the ICP group and non-ICP group, as was the disability rate at discharge. A higher peak ICP value, rather than a continuous ICP of more than 25 mmHg, was an independent predictor for perioperative death, and the cut-off value of peak ICP value for predicting mortality was found as 31 mmHg.

Whether DC can improve the prognosis of patients with severe cerebral hemorrhage has been controversial. The guideline for TBI proposed by AANS in 2016 indicated that for patients with diffuse severe TBI (without local occupying lesion) with ICP > 20 mmHg and cumulative time > 15 min/h and failing first-line treatment, dual frontal DC cannot improve prognosis, but can reduce ICP and shorten the length of intensive care unit (ICU) stay [16]. One prospective, multicenter, randomized controlled trial (RCT) confirmed that for adult patients with diffuse severe TBI and refractory intracranial hypertension, early DC can reduce ICP and shorten the length of ICU stay, but it cannot reduce the mortality of 6 months after injury, and the proportion of patients with poor prognosis (GOS score 1–4) was increased at 6 months after injury [7]. However, the study was widely questioned because of its design flaws, such as the broader inclusion criteria (ICP > 20 mmHg and cumulative time > 15 min/h), and significant baseline differences between groups, etc. Another subsequent RCT study RESCUEicp adopt different inclusion criteria based on ICP value (ICP > 25 mmHg and cumulative time > 1–12 h) and indicated that DC can reduce the mortality at 6 months and 12 months after injury, especially significantly reducing the early mortality after injury. However, the proportion of lower severe disability and upper severe disability increased, and the proportion of moderate disability and good recovery was similar with conservative management [17]. In this study of spontaneous intracerebral hemorrhage, the perioperative mortality was 20.5%. 28.4% were mild coma, 21.0% were moderate coma, and 30.1% were severe coma. 29.0% have deteriorated GCS scores at 2 weeks after the operation compared with admission.

DC is an emergency response to severe intracranial hypertension, and the control of ICP and restoration of cerebral perfusion pressure is an important content in the perioperative treatment. However, the ICP management target is still poorly understood, and even the ICP threshold in the case of cerebral hernia is still unknown [18]. In clinical practice, doctors usually need to make a comprehensive evaluation on the clinical manifestations (such as cognitive state, pupil), imaging manifestations (such as midline shift, brain edema, or swelling), ICP monitoring (if any), to decide whether DC is necessary [19]. On the other hand, postoperative intracranial pressure monitoring is helpful for clinical workers to adjust the dosage of dehydrating drugs such as mannitol in real time based on the ICP value. Moreover, real-time monitoring of ICP after operation is helpful to timely find the changes of the condition and effectively reduce the incidence of serious complications such as electrolyte disturbance and renal injury after operation [20]. However, one previous study reported no effects on ICP monitoring patients whatever mortality or functional outcome [21]. In this study, we found no difference in the perioperative prognosis between the ICP group and non-ICP group, which indicated that the management protocol beyond monitoring alone might improve the prognosis. Therefore, a ICP management target should be explored based on the association between ICP monitoring parameters and patient outcomes. One recent study proposed that neurocritical care management of patients using multimodal monitoring can improve outcomes [22]. In this study, the peak ICP value was found statistically significantly associated with perioperative death, which means that strict control of the peak ICP value may reduce perioperative mortality. In previous studies, the reported cut-off ICP for DC in refractory high ICP is different [23]. In this study, the cut-off ICP was preset to 25 mmHg. However, an ICP value > 25 mmHg was found no significant association with perioperative death. The further ROC consistency test showed the cut-off value of peak ICP value was 31 mmHg, which means keeping the peak value of ICP below 31 mmHg at all times may be more meaningful than other measures.

According to the position of the ICP monitoring probe in the cranial cavity, it can be divided into ventricular monitoring method, brain tissue monitoring method, and subdural or epidural monitoring method [24]. Intraventricular monitoring is the most accurate method. However, in clinical practice, the ventricle on the bleeding side is often compressed to become smaller or displaced, resulting in the difficulty of placing the ICP probe. Repeated puncture may lead to new intracranial hemorrhage and increase the risk of postoperative complications. In this study, the ICP monitoring probes were placed intracerebral to minimize shift and monitoring inaccuracy. However, changes in postures (sitting position or supine position) tend to lead to large variations in ICP values. One recent study confirmed that the postural ICP difference remained constant at around 8 mmHg (sitting position and supine position) [25]. In this study, we collected ICP values measured in supine position. Besides, most of the severe intracranial hemorrhage patients showed irritability during the perioperative period, which can lead to a higher ICP. Therefore, sedation and analgesia may be necessary for these patients to control ICP [22, 26–28].

### Limitations

Several potential limitations of this study should be noted. First, the small sample size might reduce the power of our conclusions. Second, it is a single-center study and selection bias existed. Operative indications, surgical procedures, and perioperative patient management can vary according to institutional philosophy and experience. The control group was derived from earlier practice. Although the time period of this cohort was relatively restricted, it might contribute to discrepant quality of care. The impact of surgical technique was not carefully assessed between groups, for example, residual hematoma after surgical evacuation, which might contribute to potential bias. Third, only the short-term prognosis at 2 weeks after operation was included as the primary goal, and the evaluation of long-term prognosis such as 6 months and 1 year were lacked. However, the intracranial condition was considered stable at 2 weeks after surgery, which could reflect the long-term prognosis. In the future, researchers should organize a large-sample multicenter RCT study on the application of ICP in spontaneous intracerebral hemorrhage.

## Conclusions

A high ICP peak value might provide a better prediction for the mortality of patients with spontaneous intracerebral hemorrhage evacuation and DC, suggesting a tailored ICP management protocol to decrease ICP peak value.

## Data Availability

The datasets used and/or analyzed during the current study are available from the corresponding author on reasonable request.

## Abbreviations

CI: Confidence interval
DC: Decompressive craniectomy
GCS: Glasgow Coma Scale
GOS: Glasgow Outcome Scale
ICP: Intracranial pressure
OR: Odds ratio
ROC: Receiver operating characteristics
